# The effects of phenoxodiol on the cell cycle of prostate cancer cell lines

**DOI:** 10.1186/s12935-014-0110-z

**Published:** 2014-11-08

**Authors:** Simon Mahoney, Frank Arfuso, Michael Millward, Arun Dharmarajan

**Affiliations:** School of Anatomy, Physiology and Human Biology, Faculty of Science, The University of Western Australia, Crawley, Perth, WA 6009 Australia; Curtin Health Innovation Research Institute, Biosciences Research Precinct, School of Biomedical Sciences, Faculty of Health Sciences, Curtin University, GPO Box U1987, 6845 Perth, WA Australia; School of Medicine and Pharmacology, The University of Western Australia, Crawley, Perth, WA 6009 Australia

**Keywords:** Phenoxodiol, Cell cycle, Prostate cancer, Cytotoxicity

## Abstract

**Background:**

Prostate cancer is associated with a poor survival rate. The ability of cancer cells to evade apoptosis and exhibit limitless replication potential allows for progression of cancer from a benign to a metastatic phenotype. The aim of this study was to investigate *in vitro* the effect of the isoflavone phenoxodiol on the expression of cell cycle genes.

**Methods:**

Three prostate cancer cell lines-LNCaP, DU145, and PC3 were cultured *in vitro*, and then treated with phenoxodiol (10 μM and 30 μM) for 24 and 48 h. The expression of cell cycle genes p21^WAF1^, c-Myc, Cyclin-D1, and Ki-67 was investigated by Real Time PCR.

**Results:**

Here we report that phenoxodiol induces cell cycle arrest in the G1/S phase of the cell cycle, with the resultant arrest due to the upregulation of p21^WAF1^ in all the cell lines in response to treatment, indicating that activation of p21^WAF1^ and subsequent cell arrest was occurring via a p53 independent manner, with induction of cytotoxicity independent of caspase activation. We found that c-Myc and Cyclin-D1 expression was not consistently altered across all cell lines but Ki-67 signalling expression was decreased in line with the cell cycle arrest.

**Conclusions:**

Phenoxodiol demonstrates an ability in prostate cancer cells to induce significant cytotoxicity in cells by interacting with p21^WAF1^ and inducing cell cycle arrest irrespective of p53 status or caspase pathway interactions. These data indicate that phenoxodiol would be effective as a potential future treatment modality for both hormone sensitive and hormone refractory prostate cancer.

## Background

Advanced prostate cancer has a 5-year survival of only 30%. Historically, chemotherapy has been used with palliative intent but unclear survival benefit for these advanced-stage patients [1]. Current practices for hormone-refractory/castrate resistant, metastatic prostate cancer incorporate the use of taxanes. Docetaxel, in particular, is being incorporated in numerous current clinical trials either as a single or combination agent against androgen-independent prostate cancer, and it is also being investigated for its use as a neoadjuvant or adjuvant agent in hormone sensitive, locally aggressive prostate cancer [2].

Two of the hallmarks of cancer are the ability to be self-sufficient in growth signals and to have infinite replicative potential, which can be initiated through damaging the cell cycle restriction points, thereby allowing for progression of cancer from benign to metastatic states [3]. The cell cycle is composed of G1, S, G2, and M phases, which represent normal function, DNA replication, organelle replication, and mitotic separation respectively [4]. Any errors that may occur at these steps could be catastrophic for normal cell functioning and, as such, the cell cycle apparatus retains potent signalling molecules that can search for errors and rapidly induce apoptosis [5]. The initial point where this process can occur is referred to as the G1 restriction point [6].

Docetaxel and paclitaxel are taxanes that bind to and stabilize microtubules, causing G2/M cell-cycle arrest and apoptosis. Although the action and anti-cancer activity of paclitaxel and docetaxel are much the same, key differences exist clinically; docetaxel shows activity in patients with metastatic solid tumors that are resistant to paclitaxel [7]. The actual mechanisms that lead to cell death remain unclear but may include activation of intrinsic pathways essential for apoptosis, induction of bcl-2 phosphorylation facilitating apoptosis, and inhibition of angiogenesis. Cell death after exposure to docetaxel appears to involve apoptotic mechanisms, including classic features such as DNA fragmentation, cell volume shrinkage, and membrane-bound apoptotic bodies [7]. Studies have also shown that apoptosis induced by taxanes involves several apoptotic signal molecules, such as JNK, protein kinase A, c-Raf-1/Ras/Bcl-2, p53/p21^WAF1^, and mitogen-activated protein kinases (ERK and p38). However, the mode of apoptotic action in different tumors is far from clear [8].

In this study, we explored the ability of phenoxodiol to impact the cell cycle of prostate cancer cells and investigate the underlying signalling pathways c-Myc, Cyclin-D1, Ki-67 and p21^WAF1^. Phenoxodiol, [2H-1-benzopyran-7-0, 1,3-(4-hydroxyphenyl)], is a synthetic isoflavone molecule first isolated from soy beans and now currently undergoing Phase III clinical trials for the treatment of platinum and taxane refractory ovarian cancer [9,10]. We examined three cell lines: LNCaP cells are responsive to 5-alpha-dihydrotestosterone (DHT), which means they contain a functioning androgen receptor (AR) and are indicative of an early stage prostate cancer cell line even though they are metastatic in nature. DU145 cells are used as a classical example of late stage prostate cancer and have moderate metastatic potential. The cell line is only weakly positive for acid phosphatase and exhibits very low DHT activity and is considered androgen receptor (AR) negative. PC3 cells are used as an example of late stage prostate cancer and exhibit high metastatic potential. It has been determined that DU145 and PC3 cells do not respond to AR stimulation and therefore have a broken pathway, but are not truly AR negative as they do express a form of the receptor, just an inactive form.

## Results

**Figure 1:**
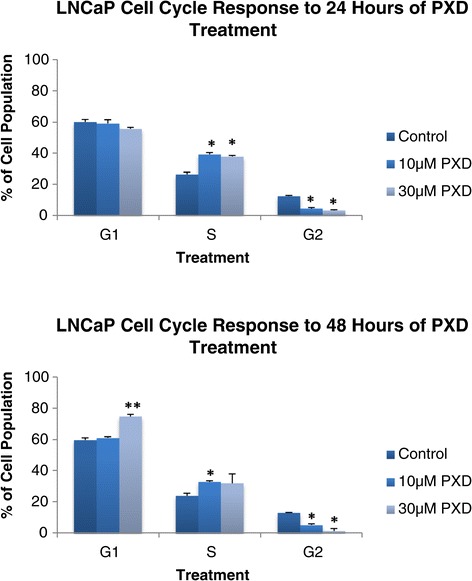
**LNCaP cycle analysis after 24 and 48 hours of 10 μM and 30 μM phenoxodiol treatment.**

We have previously determined a growth response curve for all three cell lines [11] (See Figure 1 in that paper) that was subsequently used as the basis for the treatment times and doses used in our current study. We also determined that apoptotic signalling was not consistently altered in response to phenoxodiol treatment and, therefore, it was not directly targeting one specific pathway tested [11]. Another of the hallmarks of cancer, the ability to limitlessly replicate, was investigated through looking at the cell cycle response to phenoxodiol treatment. After 24 or 48 hours of phenoxodiol treatment, cells were stained with propidium iodide and analysed to determine DNA content, which was then assessed into cell cycle phase populations G1, S, and G2 phase using FlowJo software.

### LNCaP: cell cycle analysis after 24 and 48 hours of 10 um and 30 um phenoxodiol treatment

Figure 1 demonstrates LNCaP cell line cell cycle response to 10 μM and 30 μM phenoxodiol treatment over 24 and 48 hours by assessing the cell cycle phase populations differentiated by DNA content. Phenoxodiol induced significantly decreased G2 phase cell populations versus DMSO vehicle control, over 24 hours for both 10 μM (p < 0.001) and 30 μM (p < 0.001) phenoxodiol treatments in LNCaP cells. The S phase cell population was found to increase versus DMSO vehicle control following 24 hours of 10 μM (p < 0.0021) and 30 μM (p = 0.0016) phenoxodiol treatment. The 10 μM and 30 μM phenoxodiol treatment groups were not significantly different versus each other after 24 hours.

### DU145: cell cycle analysis after 24 and 48 hours of 10 um and 30 um phenoxodiol treatment

**Figure 2:**
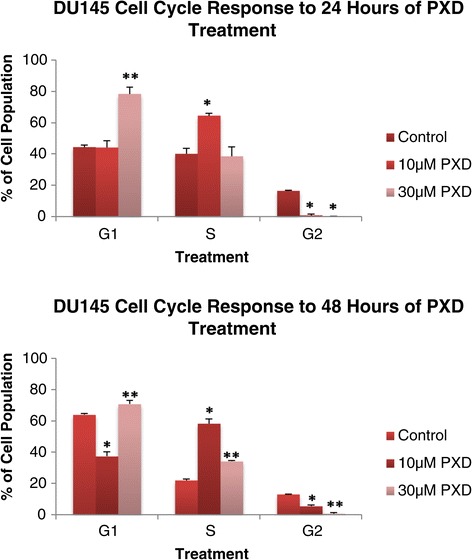
**DU145 cycle analysis after 24 and 48 hours of 10 μM and 30 μM phenoxodiol treatment.**

Figure 2 demonstrates the DU145 cell line cell cycle response to 10 μM and 30 μM phenoxodiol treatment over 24 and 48 hours by assessing the cell cycle phase populations differentiated by DNA content. Following 24 hours of phenoxodiol treatment, the DU145 cell line’s G2 phase cell population was significantly decreased versus DMSO vehicle control in both 10 μM (p = 0.0028) and 30 μM (p = 0.0021) concentrations. Only the 10 μM phenoxodiol treatment had an increased S phase cell population versus DMSO vehicle control (p < 0.001) but the 30 μM treatment had an increased G1 phase cell population versus DMSO vehicle control (p = 0.0038) and 10 μM (p = 0.0049).

Phenoxodiol induced significantly decreased G2 phase cell populations in DU145 cells over 48 hours for both 10 μM (p < 0.001) and 30 μM (P < 0.001) treatments versus DMSO vehicle control and between the treatment concentrations (P < 0.001). The S phase cell population was significantly increased versus DMSO vehicle control in both 10 μM (p < 0.001) and 30 μM (p < 0.001) treatments, as well as significantly different between treatments (p < 0.001). The G1 phase cell population was significantly decreased versus DMSO vehicle control for the 10 μM treatment (p < 0.001) and significantly increased versus DMSO vehicle control (p < 0.001) and 10 μM (p < 0.001) for the 30 μM treatment group. In DU145 cells, phenoxodiol was determined to consistently decrease G2 phase cell population over 24 and 48 hours following treatment with 10 μM and 30 μM concentrations.

### PC3: cell cycle analysis after 24 and 48 hours of 10 um and 30 um phenoxodiol treatment

**Figure 3:**
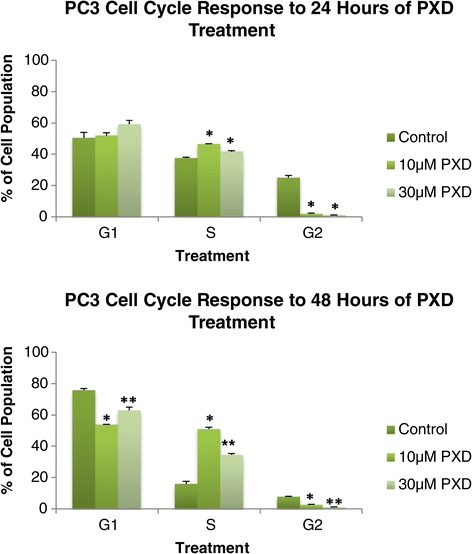
**PC3 cycle analysis after 24 and 48 hours of 10 μM and 30 μM phenoxodiol treatment.**

Figure 3 demonstrates the PC3 cell line cell cycle response to 10 μM and 30 μM phenoxodiol treatment over 24 and 48 hours by assessing the cell cycle phase populations differentiated by DNA content. Following 24 hours of phenoxodiol treatment, the PC3 cell line’s G2 phase cell population was significantly decreased versus DMSO vehicle control in both 10 μM (p < 0.001) and 30 μM (p = 0.0071) concentrations. No significant differences were detected in G1 phase cell population, but S phase cell populations were significantly increased versus DMSO vehicle control for 10 μM (p < 0.001) and 30 μM (p = 0.0022) treatments.

Following 48 hours of treatment, the PC3 G2 phase cell population was significantly decreased versus DMSO vehicle control in both 10 μM (p < 0.001) and 30 μM (p < 0.001) treatments as well as significantly different between the treatments (p < 0.001). The PC3 S phase cell population was significantly increased versus DMSO vehicle control in both 10 μM (p < 0.001) and 30 μM (p < 0.001) and significantly different between treatments (p < 0.001). The PC3 G1 phase cell population was significantly decreased versus DMSO vehicle control in both 10 μM (p < 0.001) and 30 μM (p < 0.022) treatments and significantly different between the treatments (p < 0.001). In PC3 cells, phenoxodiol was determined to consistently decrease G2 phase cell population over 24 and 48 hours following treatment with 10 μM and 30 μM concentrations.

### Quantitative PCR expression of signalling pathways following phenoxodiol treatment

Once it was determined that phenoxodiol had an impact on the cell cycle, it was necessary to explore the underlying cell cycle and cell proliferation signalling that could be the target of phenoxodiol treatment. Quantitative PCR (qPCR) analysis was performed on all three cell lines, with the housekeeping gene L19 used as a standardising agent; control expression was designated as one.

### Quantitative PCR analysis of *c-Myc*, *Cyclin D1*, *Ki-67*, and *p21*

**Figure 4:**
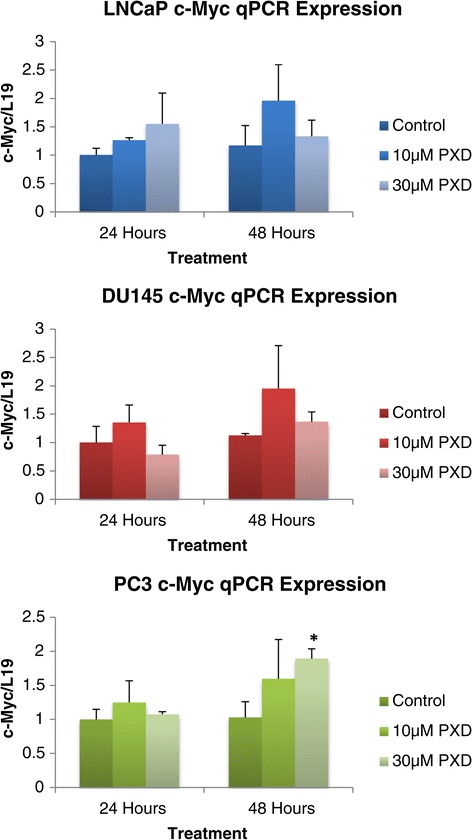
***c-Myc*** **mRNA expression analysis of prostate cancer cells over 24 and 48 hours post phenoxodiol treatment.**

*c-Myc* is a potent initiator of cell replication and has been implicated in increasing the rate at which cells enter S phase [12]. Figure 4 demonstrates the quantitative mRNA expression of *c-Myc* in cells treated over 24 and 48 hour periods with phenoxodiol. After 48 hours of 30 μM phenoxodiol treatment, PC3 cells were found to significantly increase the expression of *c-Myc* versus DMSO vehicle control (p = 0.033). Neither LNCaP nor DU145 cells were found to have any significant changes in *c-Myc* expression in response to phenoxodiol treatment.

**Figure 5:**
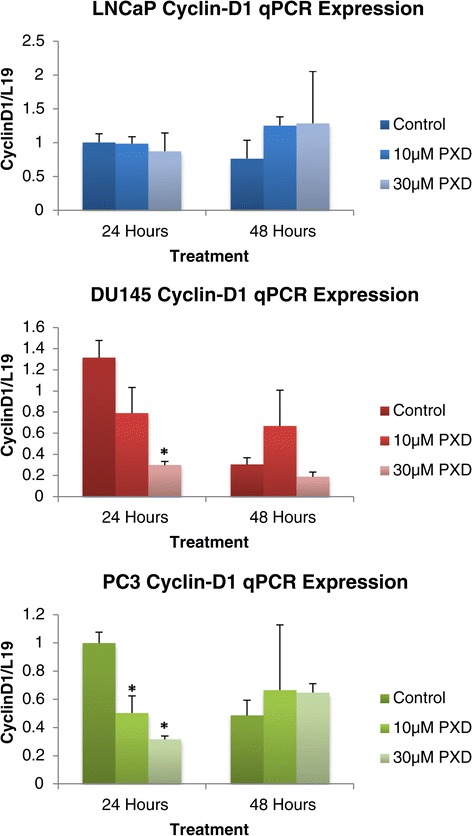
***cyclin-d1*** **mRNA expression analysis of prostate cancer cells over 24 and 48 hours post phenoxodiol treatment.**

*Cyclin-D1* is recognised as potent initiator of cell cycle progression from G1 through to S phase by the Cyclin-D1 Cdk4 complex activating the Cyclin E Cdk2 complex, which results in inhibition of the cell cycle inhibiting Rb protein [13]. Figure 5 demonstrates the quantitative mRNA expression of the cell cycle regulator gene *Cyclin-D1* over 24 and 48 hours post phenoxodiol treatment in prostate cancer cells. Decreasing *Cyclin-D1* expression, in response to phenoxodiol treatment, could result in quiescent and apoptotically sensitive cells. LNCaP cells did not have a detectable change in *Cyclin-D1* expression level under the influence of phenoxodiol treatment. DU145 cells were found to have a significant decrease in the expression of *Cyclin-D1* versus DMSO vehicle control (p = 0.0071) after 24 hours of treatment with 30 μM phenoxodiol no other changes in expression were detected in the DU145 cell line. PC3 cells exhibited a similar trend to treatment as the DU145 cells, with both the 10 μM phenoxodiol (p = 0.026) and 30 μM phenoxodiol (p = 0.0011) treatments significantly decreased in expression versus DMSO vehicle control after a 24 hour period of treatment.

**Figure 6:**
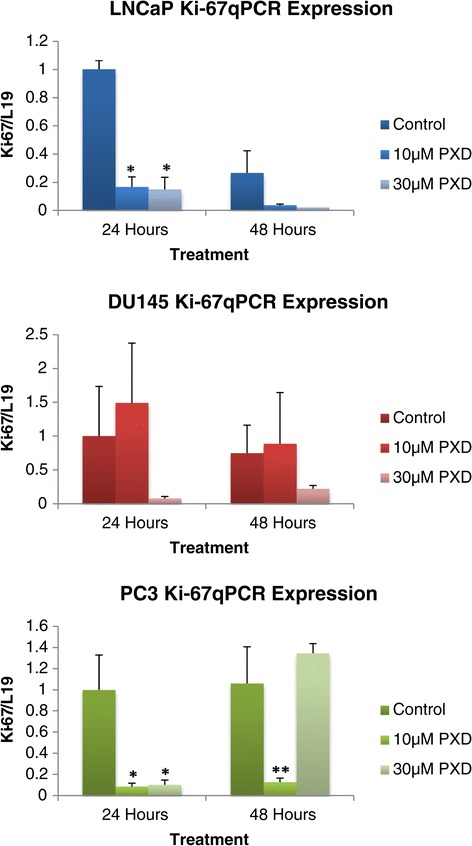
***Ki-67*** **mRNA expression analysis of prostate cancer cells over 24 and 48 hours post phenoxodiol treatment.**

*Ki-67* expression is an effective indicator of rate of proliferation in cells [14]. Figure 6 demonstrates the quantitative mRNA expression of the cell proliferation gene *Ki-67* over 24 and 48 hours post phenoxodiol treatment in prostate cancer cells. LNCaP cells were significantly decreased in *Ki-67* mRNA signalling over 24 hours with both 10 μM (p = 0.0043) and 30 μM (p = 0.0065) phenoxodiol treatments exhibiting decreased mRNA expression versus DMSO vehicle control. DU145 cells had no significant difference in *Ki-67* mRNA expression over 24 and 48 hours of treatment with 10 μM and 30 μM PXD although a biological trend towards decreased expression was indicated (p = 0.082, p = 0.1 respectively). PC3 cells exhibited a significant decrease in *Ki-67* mRNA signalling, over 24 hours, in both 10 μM (p = 0.034) and 30 μM (p = 0.036) phenoxodiol treatments while the 48 hour 10 μM phenoxodiol treatment exhibited significantly decreased mRNA expression versus DMSO vehicle control (p < 0.05) and 30 μM phenoxodiol (p = 0.032).

**Figure 7:**
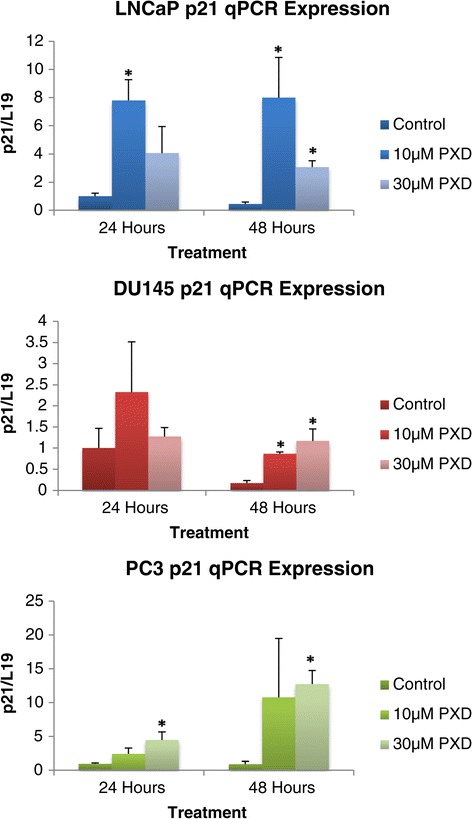
***P21*** **mRNA expression analysis of prostate cancer cells over 24 and 48 hours post phenoxodiol treatment.**

Figure 7 demonstrates the quantitative mRNA expression of the cell proliferation gene *p21*^*WAF1*^ over 24 and 48 hours post phenoxodiol treatment in prostate cancer cells. *P21*^*WAF1*^ is a cell cycle inhibiting factor that can prevent the formation of the Cyclin E Cdk2 complex. This inhibition prevents the progression of the cell through G1 to S phase [15]. LNCaP cells exhibited a significant increase in *p21* mRNA expression versus DMSO vehicle control over 24 hours of treatment with 10 μM phenoxodiol (p = 0.0099), and over 48 hours of treatment with both 10 μM (p < 0.050) and 30 μM phenoxodiol (p = 0.011) concentrations. DU145 cells exhibited a significant increase in *p21* mRNA expression versus DMSO vehicle control after 48 hours of treatment with both 10 μM (p = 0.0048) and 30 μM (p = 0.028) phenoxodiol concentrations. PC3 cells exhibited a significant increase in *p21* mRNA expression over both 24 and 48 hours of treatment (p = 0.042 and p = 0.0044 respectively) with the 30 μM phenoxodiol treatment group.

## Discussion

Two of the hallmarks of cancer are the ability to be self-sufficient in growth signals and to have infinite replicative potential, which can be initiated through damaging the cell cycle restriction points, allowing for progression of cancer from benign to metastatic [3,16]. Here we report that phenoxodiol induces cell cycle arrest in the G1/S phase of the cell cycle, with the resultant arrest due to the up regulation of *p21*^*WAF1*^. The cytotoxicity may be due to downstream signalling of molecules such as Akt and ASK1 [17-19]. *c-Myc* is a potent oncogene and expression was found to alter in PC3 cells in response to phenoxodiol. We also report that the expression of *Ki-67* and *Cyclin-D1* was altered after phenoxodiol treatment. Upon activation of mitogenic signalling, cells commit to entry into a series of regulated steps allowing completion of the cell cycle. Cells begin in G1 phase, the time between M and S phases, and before entry into S phase, where DNA is replicated, must pass through a restriction point [6] that analyses and attempts to repair DNA damage. After S phase, cells enter G2 phase (the time between the S and M phases), where cells can repair errors that occurred during DNA duplication, preventing the propagation of these errors to daughter cells. Finally, the separation into two daughter cells by chromatid separation occurs and is called M phase [20]. The sequence of events in cell cycle progression is highly orchestrated and depends on the cyclic activation and inactivation of cyclin dependent kinases (CDK), which govern the progression of the cells from one phase to another. In the event of tumourigenesis, constitutive mitogenic signalling as well as mutations in tumour suppressor genes and proto-oncogenes leads to cell cycle deregulation and uncontrolled proliferation [15,21].

The tumour suppressor *p53* is the primary controller of cell cycle activity, which triggers cell cycle arrest, induces the repair of DNA damage or apoptosis by induction of *p21*^*WAF1*^, *p53R2*, *Bax*, and *Puma* [22]. p21^WAF1^ is a cyclin dependant kinase inhibitor (CdkI) family member, along with p27 and p57, which interfere with the cyclin dependant kinase-cyclin complex. The group is regulated both by internal and external signals with the expression of p21^WAF1^ under transcriptional control of the p53 tumour suppressor gene [23]. In this study we exhibited the ability of phenoxodiol to induce cell cycle arrest at both 10 μM and 30 μM concentrations over 24 and 48 hours of treatment, with the resultant cells exhibiting significantly decreased cell populations of G2 cells and subsequent arrest of the cell cycle visible in the significant alteration of G1 and S phase cell populations. In all cell lines the decrease in G2 phase cell population was consistent and resulted in very low cell populations, while some treatments resulted in high S phase arrest and others in G1 phase arrest. It is clear that the method of phenoxodiol induced cell cycle arrest is independent of p53 status, with LNCaP (p53-wild type), DU145 (p53-mutated), and PC3 (p53-null) cells all representing different p53 status cell types.

*p21*^*WAF1*^ is a tumour suppressor gene that can induce disruption of the Cyclin-e/Cdk2 complex and prevent subsequent progression from G1 phase into S phase of the cell cycle [18,24]. Numerous studies have shown that up regulation of *p21*^*WAF1*^ causes growth arrest in various cancer models and, though *p21*^*WAF1*^ was initially identified to be transcriptionally up-regulated by *p53* in response to DNA damage, recent studies have shown that *p21*^*WAF1*^ can also be induced by various transcription factors with subsequent mediation of cell cycle arrest, senescence, and apoptosis in a *p53* independent manner [25,26]. The ability of isoflavones, and specifically phenoxodiol, to induce cell cycle arrest has been previously reported with studies indicating that arrest was induced by *p21*^*WAF1*^ stabilisation and expression increase [25,27,28]. We investigated the expression of *p21*^*WAF1*^ after the data indicated significant cell cycle arrest as a response to phenoxodiol treatment. *p21*^*WAF1*^ signalling expression was found to be significantly increased across all the cell lines in response to treatment, indicating that activation of *p21*^*WAF1*^ was occurring via a *p53* independent manner, with resulting cell cycle arrest. The induction of cytotoxicity in the cells was independent of caspase activation, as previously shown, and potentiated by induced mitotic depolarisation. This confirms previous studies that have indicated that isoflavones induce cell cycle arrest through activation and stabilisation of *p21*^*WAF1*^ [27,28].

The assembly of Cyclin-D1, with its CDK4/6 partners, is a mitogen regulated process occurring in early G1; with the resultant Cyclin-D1-CDK4/CDK6 complexes promoting G1 progression by inhibiting the activity of the retinoblastoma protein (Rb), resulting in activation of E2F and subsequent cyclin/cdk signalling, which enters the cell into the cell cycle [13,29]. Many oncogenic signals induce *Cyclin-D1* expression [30-32] and do so through distinct DNA sequences in the Cyclin-D1 promoter, including Ras, Src,ErbB2, and β-catenin. Decreasing the expression of *Cyclin-D1*, or interference with the cyclin/Cdk complex results in cell arrest in G1 phase and eventual senescence. We investigated the expression of *Cyclin-D1* after treatment with phenoxodiol and determined that DU145 and PC3 both had a significant decrease in signalling over 24 hours but not over 48, while LNCaP cells did not change expression of *Cyclin-D1* signalling. While not a direct target of phenoxodiol treatment in prostate cancer cells, the role of *Cyclin-D1* seems to be tissue and oncogene specific, with *Cyclin-D1* linked to activation of the Wnt/β-catenin signalling pathway [33].

Ki-67 antigen is present in all proliferating cells (normal and neoplastic) and its evaluation allows determination of the rate of growth. *Ki-67* expression has been shown to have a strong relationship with Gleason’s grading, which has an important correlation with the prognosis of prostate cancer and, as such, it is an independent predictive factor in both patient survival assessment and disease recurrence [14,34]. *Ki-67* expression was found to significantly decrease in LNCaP and PC3 cells, while there was a biological trend towards this in DU145 cells but error margins resulted in no detection of significant alteration. The decreased *Ki-67* signalling expression confirms the cell cycle arrest data and indicates that prostate cancer cells are undergoing senescence induced cytotoxicity. *c-Myc* signalling has been shown to be a proto-oncogene, with regulation of *c-Myc* expression inducing the expression of other oncogenes, in response and leading to a neoplastic cell type. It is known that cell cycle regulation is altered under excess *c-Myc* expression, with a decrease in time taken to reach the restriction point of G1 [12]. In this study we determined that *c-Myc* expression was only altered under the influence of phenoxodiol in PC3 cells after 48 hours of treatment, with signalling potentially being a mechanism to drive the cell out of arrest.

## Conclusions

In this study we determined that phenoxodiol treatment induced significant cell cycle arrest across 24 and 48 hours of 10 μM and 30 μM phenoxodiol treatment. *p21*^*WAF1*^ expression was found to be significantly increased across all the cell lines in response to treatment, indicating that activation of *p21*^*WAF1*^ and subsequent cell arrest was occurring via a *p53* independent manner, with induction of cytotoxicity independent of caspase activation. We determined that *c-Myc* and *Cyclin-D1* expression was not consistently altered but that *Ki-67* signalling expression was decreased in line with the cell cycle arrest.

Phenoxodiol demonstrates an ability in prostate cancer cells to induce significant cytotoxicity in cells by interacting with *p21*^*WAF1*^ and inducing cell cycle arrest irrespective of *p53* status or caspase pathway interactions. These data indicate that phenoxodiol would be effective as a potential future treatment modality for both hormone sensitive and hormone refractory prostate cancer.

## Materials and methods

### Cell culture

Cells were cultured as previously published [11]. LNCaP (ATCC: CRL-1740), DU145 (ATCC: HTB-81), and PC3 (ATCC: CRL-1435) human prostate cells were all grown with 10% FBS, 2 mM L-glutamine and 1% penicillin/streptomycin media in a 5% CO_2_ atmosphere at 37°C. LNCaP cells were grown in RPMI 1640 media (Gibco) supplemented with 10 mM HEPES, 2.5 g/l glucose and 1 mM sodium pyruvate. DU145 cells were grown in MEM with Earle’s BSS media (Gibco) supplemented with 1 mM sodium pyruvate and 0.1 mM Non-Essential Amino Acids. PC3 cells were grown in Hams F12K media (Gibco).

### Cell cycle analyses

Analysing the population of cells in each cell cycle phase indicated whether phenoxodiol had a direct effect upon the cell cycle of prostate cancer cells. Briefly, cells were seeded into 6 well plates and incubated for 48 hours before 24 or 48 hours of treatment media were applied. Cell media were aspirated and centrifuged and cells were trypsinised, then placed into the same tube and centrifuged. Cells were washed with PBS and then -20°C 70% ethanol was added drop wise, while vortexing to fixate the cells. Cells were stored at 4°C then rehydrated with PBS and stained with propidium iodide before being placed into a FACS Canto II cytometer, which determined fluorescence and therefore DNA content. FlowJo software was utilised to further analyse the sample and distinguish quantitative populations of G1, S and G2 phase cells.

### Phenoxodiol

Phenoxodiol (Sigma-Aldrich Cat#D7446) was made as a stock solution of 10 mg/ml in DMSO and stored at -20°C, protected from light. The stock was used within 7 days. The stock solution was diluted 100-fold in growth medium as a working dilution. This was then further diluted in growth medium to a final concentration of 10 μM or 30 μM. Vehicle control and 10 μM phenoxodiol treatment were augmented accordingly with DMSO to ensure uniform concentration.

### Reverse transcription and quantitative RT-PCR

Cells were seeded and treated as described for DNA 3′-end analysis. Cells floating in supernatant were centrifuged and combined with adherent cells per well. RNA was isolated using TriReagent (Astral Scientific) according to manufacturer’s instructions then treated with DNA-*Free*™ (Ambion) according to manufacturer’s instructions, to remove contaminating genomic DNA. One microgram of RNA was reverse transcribed using M-MLV (Promega) and random primers (Promega) according to manufacturer’s instructions. The resulting cDNA was purified (MoBio PCR clean-up kit) prior to real-time quantitative RT-PCR. Primers used to amplify *AIF, BAX, Bcl-xL, Caspase-3, xIAP*, and *L19* (housekeeping gene) are outlined in Table 1. RT-PCR was performed in 10 μl reactions using iQ SYBR green supermix (Biorad) and 1 μl cDNA in the Rotor-Gene 3000 (Corbett Research). Cycling conditions were 95°C (0-45 s), annealing temperature as per Table 1 (15-45 s) and 72°C (5-45 s) for 45 cycles. Amplification was checked by melt curve analysis and by electrophoresis in 2% agarose/ethidium bromide for product size. Threshold cycle values for cDNA samples were compared against a standard curve obtained by amplification of 10-fold dilutions of corresponding PCR product. Each cDNA sample was run in duplicate and an average obtained. All mRNA levels were normalized by dividing by L19 mRNA levels for each cDNA sample.

**Table 1:** **Primer sequences, product size, and annealing temperature**

**Gene**	**Sequence**	**Product size (base pairs)**	**Annealing temperature**
**AIF**	*Forward*: GATCACGCTGTTGTGAGTGG	179 bp	61°C
	*Reverse*: TCTTGTGCAGTTGCTTTTGC		
**β-Catenin**	*Forward*: GATTTGATGGAGTTGGAC	218 bp	52°C
	*Reverse*: TGTTCTTGAGTGAAGGAC		
**Bax**	*Forward*: GCTGGACATTGGACTTCCTC	167 bp	61°C
	*Reverse*: TCAGCCCATCTTCTTCCAGA		
**Bcl-xL**	*Forward*: ACAATGCAGCAGCCGAGAG	167 bp	61°C
	*Reverse*: ATGTGGTGGAGCAGAGAAGG		
**Caspase 3**	*Forward:* AAGGATCCTTAATAAAGGTATCCATGGAGAACACT	322 bp	55°C
	*Reverse*: AAAGAATTCCATCACGCATCAATTCCACAATTTCTT		
**Cyclin-D1**	*Forward*: AACTACCTGGACCGCTTCCT	165 bp	62°C
	*Reverse*: CCACTTGAGCTTGTTCACCA		
**GAPDH**	*Forward*: CAGAACATCATCCCTGCATCCACT	185 bp	60°C
	*Reverse*: GTTGCTGTTGAAGTCACAGGAGAC		
**Ki-67**	*Forward*: AGTCAGACCCAGTGGACACC	225 bp	60°C
	*Reverse*: TGCTGCCGGTTAAGTTCTCT		
**L19**	*Forward*: CTGAAGGTCAAAGGGAATGTG	194 bp	52°C
	*Reverse*: GGACAGAGTCTTGATGATCTC		
**p21** ^**WAF1**^	*Forward*: CCGAAGTCAGTTCCTTGTGG	333 bp	61°C
	*Reverse*: AAGTCGAAGTTCCATCGCTCA		
**sFRP4**	*Forward*: CGATCGGTGCAAGTGTAAAA	181 bp	56°C
	*Reverse*: GACTTGAGTTCGAGGGATGG		
**XIAP**	*Forward*: GGGGTTCAGTTTCAAGGACA	183 bp	56°C
	*Reverse*: CGCCTTAGCTGCTCTTCAGT		

### Data presentation and statistics

Data are presented as mean ± SEM. For each experiment *n* is indicated in figure legends and refers to the number of well replicates per group. Differences between control and treatment group means were measured by 2-sample t-tests. Statistical significance is p <0.05.
